# Cancer has overtaken cardiovascular disease as the commonest cause of death in Scottish type 2 diabetes patients: A population‐based study (The Ayrshire Diabetes Follow‐up Cohort study)

**DOI:** 10.1111/jdi.13067

**Published:** 2019-07-25

**Authors:** Andrew Collier, Carron Meney, Mario Hair, Lyall Cameron, James G Boyle

**Affiliations:** ^1^ Diabetes Day Center University Hospital Ayr UK; ^2^ NHS Ayrshire and Arran University Hospital Ayr UK; ^3^ Primary Care Quality and Development NHS Ayrshire and Arran Ailsa Hospital Ayr UK; ^4^ Glasgow Royal Infirmary Glasgow UK

**Keywords:** Cancer, Mortality risk, Type 2 diabetes

## Abstract

**Aims/Introduction:**

The increased mortality risk associated with diabetes is well established. The aim of the present study was to determine the causes of death of people with type 2 diabetes in Ayrshire and Arran, Scotland, between 2009 and 2014, and compare them with the national mortality rates.

**Materials and Methods:**

The primary causes of death were collated. The causes of death were clustered into nine categories: heart disease, stroke, infection, renal failure, respiratory disorders, cancer, mental health, decompensated diabetes and other. The total rates were compared with national rates using the standardized mortality ratio (SMR), and then individually with heart disease, cerebrovascular disease and cancer.

**Results:**

There were 2116 deaths with the SMR, and 145 of those were caused by type 2 diabetes (*n* = 16,643; 95% confidence interval 139–152; *P* < 0.01). The SMR was >100 in all age bands, particularly in the younger age bands (*P* < 0.01). The SMR was consistently higher for women (*P* < 0.01). The SMR for heart disease was significantly >100 for both sexes in all age bands <65 years (*P* < 0.05). There was no difference in mortality causes related to the duration of diabetes. The most common cause of death was cancer (27.8%), followed by heart disease (24.1%). The SMR for cancer deaths was significantly elevated in women (120, 95% CI 104–137; *P* < 0.05).

**Conclusions:**

This study confirmed increased mortality risk in type 2 diabetes patients, and suggests that where cardiovascular risk factors are being treated aggressively, cancer takes on a greater importance in the cause of death. Should greater consideration now be given for cancer as a complication of diabetes?

## Introduction

The increase in mortality risk associated with diabetes is well established^1^, ^2^, ^3^, ^4^, ^5^, ^6^, ^7^, ^8^, ^9^, ^10^, ^11^, ^12^, ^13^, ^14^, ^15^, ^16^. Mortality rates among people with type 2 diabetes compared with the general population appear to vary significantly depending on the glycemic control, age, duration of diabetes, renal complications and ethnicity^1^, ^2^, ^3^, ^4^, ^5^, ^6^, ^7^, ^8^, ^9^, ^10^, ^11^, ^12^, ^13^, ^14^, ^15^, ^16^. The ultimate goal of the treatment of patients with type 2 diabetes is to improve the quality of life and to reduce the mortality rate to a level comparable to individuals without diabetes. The patterns of mortality appear to be changing, and a significant decline in mortality from cardiovascular disease (CVD) in both the general and type 2 diabetes populations has been reported^3^, ^4^, ^5^, ^6^, ^7^, ^8^, ^9^, ^10^, ^11^, ^12^, ^13^, ^14^, ^15^, ^16^. The excess in mortality among type 2 diabetes patients in the past has been mainly attributable to cardiovascular disease^1^, ^2^, ^3^, ^4^, ^5^, ^6^, ^7^, ^8^, ^9^, ^10^, ^11^, ^12^, ^13^, ^14^, ^15^, ^16^, particularly myocardial infarction. The Steno‐2 Study, carried out from 1993 to 2010^10^, ^11^, plus numerous other studies^1^, ^2^, ^3^, ^4^, ^5^, ^6^, ^7^, ^8^, ^9^, ^12^, ^13^, ^14^, ^15^, ^16^ have shown that aggressive multifactorial cardiovascular treatment of type 2 diabetes patients significantly reduces the risk of CVD and death. Furthermore, a number of studies suggest that the gap between the diabetes and non‐diabetes populations is narrowing^2^, ^3^, ^4^, ^5^, ^6^, ^7^, ^8^, ^9^, ^10^, ^11^, ^12^, ^13^, ^14^, ^15^, ^16^. Improved diabetes‐related medical care plus more aggressive treatment strategies based on targeting multiple risk factors have now been recommended and implemented in the clinical care of people with type 2 diabetes^17^, ^18^, ^19^.

The aims of the present study were to investigate the age profile of the deaths among people with type 2 diabetes in Ayrshire and Arran, Scotland. The study compared the deaths with the standardized mortality ratio (SMR), the expected and actual number of deaths over the 5 years, and the standardized mortality ratio for each age band. We also investigated specific causes (heart disease, stroke and cancer) for men and women in each age banding. In addition, we determined the effect of the duration of diabetes on mortality risk (<5 years, 5–10 years and >10 years).

## Methods

A total of 46 out of 55 general practices in National Health Service (NHS) Ayrshire and Arran, covering 85% of the total patient population (aged >18 years) of Ayrshire and Arran, contributed data from their practice computer systems. Data were provided both in 2009 and in 2014. There was no significant difference in the prevalence of diabetes between practices that did and did not provide data (5.5% vs 5.7%, χ^2^ = 3.3, *P* = 0.07).

For the period under investigation, all conditions were coded in accordance with the International Classification of Diseases, 10th Revision^20^. The General Practice EMIS data were linked to the national death records by the Information Services Division of NHS National Services Scotland. Generation of the linked dataset was approved by the Clinical Governance Department, NHS Ayrshire and Arran, and Caldicott Guardian approval was obtained from each general practice. The causes of death were attributed to one of nine categories: heart disease, stroke, infection, renal failure, respiratory disorders, cancer, mental health, decompensated diabetes and other.

The duration of diabetes was calculated as the time between diagnosis and either death or the study end date. SMR was calculated from Scottish mortality rates over the same period (estimated from National Records of Scotland Vital Events Reference Tables 2015) using the same age profile as the cohort. SMR confidence intervals were calculated using Byar's method, which is accurate for observed frequencies of five or more^21^. For smaller observed frequencies, an exact method based on the Poisson distribution was used^22^. National data only gives mortality rates from specific causes (heart disease, cerebrovascular accidents and cancer) for each sex separately, so comparisons with national data are shown separately for men and women.

## Results

At the start of the study period in 2009, there were 10,679 people with type 2 diabetes in the cohort. At the end of the study in 2014, 1,764 people had died, giving a mortality rate over the 5‐year period of 165.2 per 1,000 people. By comparison, the Scottish mortality rate over the same period, using the same age profile, was estimated as 113.7 per 1,000 patients. Hence, the SMR for people with type 2 diabetes in the cohort was 145. The SMR for people with type 2 diabetes was >100 (aged >35 years) and was greater in the earlier age bands, decreasing as age increased. Table 1 also shows separate SMRs for men and women. The SMR was consistently higher for women.

**Table 1:** Standardized mortality ratio for type 2 diabetes patients over the 5‐year study period by age

Age (years)	No. deaths/sample size	SMR (95% CI†) All‡	SMR (95% CI†) Men	SMR (95% CI†) Women
<25	0/6			
25–34	2/88	542 NS (61–1,806)		1,933 (242–7,224)
35–44	14/495	330 (180–553)	157 NS (51–365)	683 (312–1,294)
45–54	60/1547	232 (177–299)	190 (133–264)	283 (182–422)
55–64	203/2725	188 (163–216)	159 (132–189)	220 (174–275)
65–74	563/3319	176 (162–192)	154 (138–172)	195 (171–222)
75–84	659/2047	132 (122–143)	124 (111–137)	137 (122–153)
≥85	263/452	102 NS (90–115)	108 NS (89–128)	96 NS (81–113)
Overall	1764/10679	145 (139–152)	136 (128–145)	148 (138–159)

^†^The 95% confidence intervals (CI) were calculated using Byar's method^1^, which is accurate for observed frequencies of five or more. For smaller observed frequencies, an exact method based on the Poisson distribution is used^21^, ^22^.

^‡^Scottish mortality rates estimated from National Records of Scotland Vital Events Reference Tables 2015 Table 5.1(b): Death rates, by sex and age, Scotland, 2001 to 2014 https://www.nrscotland.gov.uk/statistics-and-data/statistics/statistics-by-theme/vital-events/general-publications/vital-events-reference-tables/2015/section-5-deaths (accessed 15/11/17). NS, not significant.

### Specific causes of death

The distribution of cause of death by sex is shown in Table 2. The main cause of death was cancer (28%), followed by heart disease (24%). These two categories accounted for over half of all deaths. There was a statistically significant difference by sex (*P* = 0.02), with more male deaths from cancer and heart disease, and more female deaths in all other categories.

**Table 2:** Cause of death for patients with type 2 diabetes by sex

Cause of death	Men	Women	Total
Cancer	29.6% (342)	25.6% (246)	27.8% (588)
Heart disease	25.7% (297)	22.1% (212)	24.1% (509)
Respiratory	12.4% (143)	13.8% (133)	13.0% (276)
Stroke	7.6% (88)	9.6% (92)	8.5% (180)
Sepsis	5.0% (58)	7.0% (67)	5.9% (125)
Decompensated diabetes	4.2% (48)	4.7% (45)	4.4% (93)
Mental health	3.1% (36)	5.1% (49)	4.0% (85)
Renal failure	2.3% (27)	2.8% (27)	2.6% (54)
Accident/liver/neurological/other	10.0% (116)	9.4% (90)	9.7% (206)
Total	1,155	961	2,116

#### Heart disease

The SMR for patients with type 2 diabetes deaths from heart disease was >100 for both sexes in all age bands (>35 years), except the oldest (≥85 years). In men, however, the increase in SMR was not significant in the 35–54 years age groups. SMR's generally decreased with age and were consistently higher for women (Table 3).

**Table 3:** Standardized mortality ratio for heart disease, stroke and cancer by age band and sex for patients with type 2 diabetes

Men with type 2 diabetes		SMR (95% CI†) Heart disease‡	SMR (95% CI†) Stroke§	SMR (95% CI†) Cancer¶
Age (years)	*n*			
<25	1			
25–34	51			
35–44	275	331 NS (8–1,857)		
45–54	921	143 NS (46–332)	362 NS (40–1,204)	237 (118–422)
55–64	1,624	199 (133–286)	337 (161–618)	118 NS (83–163)
65–74	1,818	199 (157–249)	210 (135–313)	132 (109–158)
75–84	992	141 (110–177)	106 NS (70–154)	99 NS (80–120)
≥85	186	78 NS (46–124)	59 NS (27–112)	49 (28–82)
Overall	5,868	154 (133–177)	129 (101–163)	109 NS (97–123)

Women with type 2 diabetes		SMR (95% CI) Heart disease‡	SMR (95% CI) Stroke§	SMR (95% CI) Cancer¶
Age (years)	*n*			
<25	5			
25–34	37			
35–44	220	1,933 (242–7,224)		233 NS (6–1299)
45–54	626	1,065 (272–2,560)	266 NS (6–1,392)	109 NS (29–276)
55–64	1,101	596 (308–1,040)	347 (112–803)	114 NS (70–176)
65–74	1,501	382 (268–529)	263 (158–410)	174 (140–213)
75–84	1,055	214 (163–277)	122 NS (82–174)	92 NS (70–117)
≥85	266	92 NS (56–142)	83 NS (51–126)	89 NS (56–133)
Overall	4,811	217 (182–258)	129 (101–161)	120 (104–137)

^†^The 95% confidence intervals (CI) calculated using Byar's method^1^, which is accurate for observed frequencies of five or more. For smaller observed frequencies, an exact method based on the Poisson distribution is used^21^, ^22^.

^‡^Scottish mortality rates estimated from National Records of Scotland Vital Events Reference Tables 2015: Death rates, by sex and age, Scotland, 2001 to 2014 from ischemic heart disease (Table 6.8) https://www.nrscotland.gov.uk/statistics-and-data/statistics/statistics-by-theme/vital-events/general-publications/vital-events-reference-tables/2015/section-6-deaths-causes (accessed 16/11/17).

^§^Scottish mortality rates estimated from National Records of Scotland Vital Events Reference Tables 2015: Death rates, by sex and age, Scotland, 2001 to 2014 from cerebrovascular disease (Table 6.9) https://www.nrscotland.gov.uk/statistics-and-data/statistics/statistics-by-theme/vital-events/general-publications/vital-events-reference-tables/2015/section-6-deaths-causes (accessed 16/11/17).

^¶^Scottish mortality rates estimated from National Records of Scotland Vital Events Reference Tables 2015: Death rates, by sex and age, Scotland, 2001 to 2014 from malignant neoplasms (all sites) (Table 6.6) https://www.nrscotland.gov.uk/statistics-and-data/statistics/statistics-by-theme/vital-events/general-publications/vital-events-reference-tables/2015/section-6-deaths-causes (accessed 16/11/17). NS, not significant; SMR, standardized mortality ratio.

#### Stroke

The number of deaths from stroke was small. The SMRs for people with type 2 diabetes deaths from stroke was significantly >100 only for ages 55–74 years for both men and women. SMRs generally decreased with age, and the overall SMRs were similar for both men and women (Table 3).

#### Cancer

The overall SMR for men with type 2 diabetes was not significantly different from the national population. For women with type 2 diabetes, the overall SMR was higher. Within the age bands, there was no clear pattern, but for both men and women the SMRs for the 65–74 years age band were significantly higher. The SMR for men aged >85 years was below the national population (Table 3).

### Effect of duration of type 2 diabetes on SMR

Within the study period (2009–2014), 12.7% (2116) of people with type 2 diabetes died, this included 352 patients who were newly diagnosed within the study period: mortality was 10.1% (632) for those with duration of type 2 diabetes of <5 years; mortality was 14.7% (786) for those with duration of type 2 diabetes of 5–10 years and 13.9% (698) for those with duration of >10 years. However, causes of death were broadly similar across all three duration groups, and there were no significant differences in cause of death by duration (*P* = 0.07; Table 4).

**Table 4:** Cause of death by duration of diabetes diagnosis

Cause of death	Duration of DM diagnosis			Total
	<5 years	5–10 years	≥10 years	
Cancer	31.3% (198)	29.0% (228)	23.2% (162)	27.8% (588)
Heart disease	22.5% (142)	25.2% (198)	24.2% (169)	24.1% (509)
Respiratory	14.9% (94)	11.5% (90)	13.2% (92)	13.0% (276)
Stroke	7.1% (45)	8.7% (68)	9.6% (67)	8.5% (180)
Sepsis	4.9% (31)	6.2% (49)	6.4% (45)	5.9% (125)
Decompensated diabetes	3.5% (22)	3.9% (31)	5.7% (40)	4.4% (93)
Mental health	4.1% (26)	3.9% (31)	4.0% (28)	4.0% (85)
Renal failure	1.7% (11)	2.5% (20)	3.3% (28)	2.6% (54)
Accident/liver/neurological/other	10.0% (63)	9.0% (71)	10.3% (72)	9.7% (206)
Total	632	786	698	2116

DM, diabetes.

National mortality data were available for three categories: cancer, stroke and heart disease. Table 5 shows SMR by sex and diabetes duration for these causes, as well as all‐cause SMR based on the age profile of the duration groups. SMRs for men were higher for those with duration of diabetes of <10 years for all causes except stroke. For heart disease, the SMR for those with duration of diabetes between 5 and10 years was 175. SMRs for women were again highest in those with diabetes between 5 and10 years for all causes except heart disease. The SMR for women with type 2 diabetes from heart disease was high for all diabetes durations, rising from 154 for those with diabetes for <5 years, to 214 for those with diabetes for >10 years.

**Table 5:** Standardized mortality ratio by all causes and specific mortalities

Duration of DM diagnosis	All causes† (95% CI) *n* = 2116	Heart disease‡ (95% CI) *n* = 404	Stroke§ (95% CI) *n* = 180	Cancer¶ (95% CI) *n* = 588
Men				
<5 years	120 (107–1,330)	132 (103–167)	114 NS (73–172)	115 NS (95–137)
5–10 years	138 (125,151)	175 (143–212)	123 NS (82–176)	113 NS (94–135)
10 years	106 NS (96–117)	118 NS (94–147)	126 NS (88–175)	75 (61–92)
Women				
<5 years	130 (116–146)	154 (107–212)	98 NS (62–149)	108 NS (85–134)
5–10 years	143 (129–159)	195 (146–255)	148 (105–202)	126 (103–154)
10 years	120 (107–134)	214 (165–274)	107 NS (73–153)	78 (60–99)

^†^Scottish mortality rates estimated from National Records of Scotland Vital Events Reference Tables 2015 Table 5.1(b): Death rates, by sex and age, Scotland, 2001 to 2014 https://www.nrscotland.gov.uk/statistics-and-data/statistics/statistics-by-theme/vital-events/general-publications/vital-events-reference-tables/2015/section-5-deaths (accessed 15/11/17).

^‡^Scottish mortality rates estimated from National Records of Scotland Vital Events Reference Tables 2015: Death rates, by sex and age, Scotland, 2001 to 2014 from ischemic heart disease (Table 6.8) https://www.nrscotland.gov.uk/statistics-and-data/statistics/statistics-by-theme/vital-events/general-publications/vital-events-reference-tables/2015/section-6-deaths-causes (accessed 16/11/17).

^§^Scottish mortality rates estimated from National Records of Scotland Vital Events Reference Tables 2015: Death rates, by sex and age, Scotland, 2001 to 2014 from cerebrovascular disease (table 6.9) https://www.nrscotland.gov.uk/statistics-and-data/statistics/statistics-by-theme/vital-events/general-publications/vital-events-reference-tables/2015/section-6-deaths-causes (accessed 16/11/17).

^¶^Scottish mortality rates estimated from National Records of Scotland Vital Events Reference Tables 2015: Death rates, by sex and age, Scotland, 2001 to 2014 from malignant neoplasms (all sites) (Table 6.6) https://www.nrscotland.gov.uk/statistics-and-data/statistics/statistics-by-theme/vital-events/general-publications/vital-events-reference-tables/2015/section-6-deaths-causes (accessed 16/11/17).

95% confidence intervals calculated using Byar's method, which is accurate for observed frequencies of five or more. For smaller observed frequencies, an exact method based on the Poisson distribution is used^21^, ^22^. DM, diabetes; NS, not significant.

## Discussion

The present study confirmed that all‐cause mortality for people with type 2 diabetes remains higher than in the non‐diabetes population. The SMR for women with type 2 diabetes was 148, and for men with type 2 diabetes was 136. These SMR results are very similar to the relative risk of mortality associated with type 2 diabetes compared with a diabetes‐free population from a Scottish study published in 2016^23^. The SMR in the present study was greater in the earlier age bands, decreasing as age increased, and was consistently higher in women than men. The commonest cause of death in our cohort was cancer, followed by heart disease and then respiratory conditions. The duration of diabetes did not impact on the causes of death.

The evidence base for managing type 2 diabetes and preventing complications has improved greatly^1^, ^2^, ^3^, ^4^, ^5^, ^6^, ^7^, ^8^, ^9^, ^10^, ^11^, ^12^, ^13^, ^14^, ^15^, ^16^. Aggressive cardiovascular risk factor management, glycemic control studies and smoking cessation have shown better cardiovascular outcome^1^, ^2^, ^3^, ^4^, ^5^, ^6^, ^7^, ^8^, ^9^, ^10^, ^11^, ^12^, ^13^, ^14^, ^15^, ^16^. In addition, there has been significant improvement in diabetes management in Scotland and the rest of the UK due to the Quality and Outcomes Framework payments made to general practitioners for the management of type 2 diabetes, hypertension, dyslipidemia and other chronic diseases^24^. Furthermore, the management of type 2 diabetes in general practice is very much guideline‐driven^17^, ^18^, ^19^.

The recent Asia Pacific Cohort Studies Collaboration meta‐analysis^25^ involving nearly 1 million participants showed that after controlling for major vascular risk factors, diabetes roughly doubled the risk for occlusive vascular mortality among men, but tripled the risk among women. That meta‐analysis^25^ was consistent with previous studies showing that increased mortality in type 2 diabetes was mainly attributable to cardiovascular disease^1^, ^2^, ^3^, ^4^, ^5^, ^6^, ^7^, ^8^, ^9^, ^10^, ^11^, ^12^, ^13^, ^14^, ^15^, ^16^. Unlike the present study, that meta‐analysis was undertaken before the widespread use of statin therapy and antihypertensive medications^26^. The researchers found that diabetes conferred an increased risk of mortality for all age groups studied, particularly in women. The risk conferred by diabetes was especially high among women aged 35–59 years, with a nearly sixfold higher occlusive vascular death rate in this age group. Absolute vascular death rates were higher for men with diabetes than for women with diabetes. As women without diabetes have the best prognosis, diabetes conferred a higher relative risk among women than among men. In that meta‐analysis^26^, the death relative risks for cancer mortality were considerably smaller (relative risk 1.17) than for vascular causes, and did not differ between men and women with diabetes.

Cancer is the second leading cause of death worldwide^26^, and is recognized in the UK and USA to be more common in men than women^27^. The present study is the first study to show that cancer is the major contributing cause of the increase in all‐cause mortality seen in type 2 diabetes patients in the UK. In men with type 2 diabetes, the increase in cancer SMR was not significantly greater than in men without diabetes. In women with type 2 diabetes, the increase in cancer SMR was significantly greater than in women without diabetes. This might reflect both an increase in cancer prevalence and an improvement in the management and outcome of cardiovascular disease in women with type 2 diabetes.

Obesity, type 2 diabetes and cancer appear to be linked^28^, ^29^. A recently published meta‐analysis, including 20 million individuals, showed that diabetes is a risk factor for all‐site cancer for both men and women^28^, ^29^, and the excess risk of cancer, as in the present study, was greater for women than men^29^. Type 2 diabetes and cancer have many modifiable risk factors in common, including obesity, physical activity, diet, alcohol, smoking and long latency periods before clinically manifesting^28^, ^29^. Type 2 diabetes appears to be an independent risk factor for pancreatic, endometrial, liver, colorectal, bladder and breast cancer^29^. Possible mechanisms linking diabetes with cancer include hyperglycemia and hyperinsulinemia (endogenous or exogenous), plus alterations of the insulin‐like growth factor system, chronic subclinical inflammation, abnormalities in sex hormone metabolism, adipokines and possibly antidiabetes medication used in the management of type 2 diabetes^28^, ^29^, ^30^, ^31^, ^32^, ^33^, ^34^, ^35^, ^36^. In addition, hyperglycemia might induce oxidative stress, which could promote the formation and expression of advanced glycation products and their receptors. This interaction could activate numerous cell signaling pathways, which promote carcinogenesis and cell invasion^35^, ^36^. Furthermore, through multiple cellular signaling cascades, enhanced insulin and insulin‐like growth factor could promote cell proliferation and growth^36^. The sex differences for the associations of diabetes and some cancers (e.g., gastrointestinal) might be shown through several alternative underlying mechanisms. The sex differences could be attributable to poorer glycemic control in women^28^, ^36^, longer period of “prediabetes” in women^28^, ^36^ and various sex hormone‐binding globulins, which might affect the bioavailability of estrogen in both sexes and bioavailable testosterone in women^28^, ^29^. In addition, the increased mortality as a result of cancer in type 2 diabetes patients has been attributed to the cancer treatments, underlying disease, antidiabetes medication, plus acute and chronic complications of diabetes, such as renal and cardiovascular diseases^28^.

Mortality rates and causes of death in patients with type 2 diabetes vary according to ethnicity. The mortality caused by CVD, which was the leading cause of death among diabetes patients in the USA, declined by 32% every 10 years among people with type 2 diabetes^8^, ^9^, ^15^, ^16^. The rate of decline of CVD death was significantly greater among those with type 2 diabetes than those without diabetes^8^. Approximately 70% of the participants in that study were non‐Hispanic white people, and the decrease of the death rate due to CVD was consistent with the results of studies undertaken in white people reported in other developed countries^1^, ^2^, ^3^, ^4^, ^5^, ^6^, ^7^, ^8^, ^9^, ^10^, ^11^, ^12^, ^13^, ^14^, ^15^, ^16^. In Japan, however, the proportion of total deaths from cancer in patients with type 2 diabetes has continued to rise and exceeds that from vascular causes (proportion of deaths in patients with diabetes in 2001–2010; vascular disease 14.9%, cancer 38.3%)^15^, ^16^. This increase in cancer death among type 2 diabetes patients has continued, despite the proportion of total deaths due to cancer in the general population slightly decreasing over this period^15^, ^16^.

One of the major strengths of the present study was the accuracy of the general practice data collected. Limitations in using death certificate data are well recognized with most of the attention being focused on whether or not the death certificates in people with diabetes refer to the diabetes^36^, ^37^. In the present study we knew which individuals had type 2 diabetes and therefore diabetes did not have to appear on the death certificate. The present study had a number of limitations. The study was not large enough to investigate the types of cancer associated with type 2 diabetes. In addition, we were unable to determine the pre‐morbid body mass index, level of glycemic control, the diabetes complications plus the antidiabetic medication that the patients were taking.

Understanding the primary cause of excess mortality in type 2 diabetes patients is important in order to determine the interventions needed to decrease mortality rates. Further intervention is still required to reduce CVD death in both men and women. Why cancer death is more common in women with type 2 diabetes compared with women without diabetes is not clear and requires further investigation. Obesity appears to increase the risk of both diabetes and cancer, making lifestyle change even more important. Should greater consideration be given for cancer as a complication of diabetes and screening integrated into the follow up of patients with type 2 diabetes, particularly women? The present study was undertaken in a relatively deprived area of Scotland, where the uptake of antihypertensive medication and statin therapy is high^38^, and the population predominantly Caucasian (>98%). Further work is required to investigate the links between diabetes and cancer, types of cancer, and the role of modifiable and non‐modifiable risk factors, including hyperinsulinemia, hyperglycemia, antidiabetes medication, obesity and ethnicity.

## Disclosure

AC has received speaker fees from Sanofi, Novo, Lilly, AstraZeneca, Norgine and Bayer, and educational grants from Lilly, AstraZeneca, Norgine and Bayer. CM has received speaker fees from Sanofi, and a travel grant from Sanofi and AstraZeneca. JB has received speaker fees from Sanofi, Novo, Lilly and AstraZeneca. The other authors declare no conflict of interest.
